# Storage and initial processing of water samples for organic carbon analysis in runoff

**DOI:** 10.1016/j.mex.2020.101012

**Published:** 2020-07-26

**Authors:** Gunasekhar Nachimuthu, Mark D Watkins, Nilantha Hulugalle, Lloyd A Finlay

**Affiliations:** a NSW Department of Primary Industries, Australian Cotton Research Institute, 21888 Kamilaroi Highway, Narrabri, NSW 2390, Australia; b Lion Dairy & Drinks, Morwell, VIC, Australia; c Fenner School of Environment & Society, College of Science, Building 141, Linnaeus Way, Australian National University, Canberra, ACT 2601, Australia; d Narrabri Shire Council, Narrabri, NSW 2390, Australia

**Keywords:** Water quality, Storage time, Freezing and thawing, Dissolved organic carbon

## Abstract

Runoff monitoring experiments are often conducted in remote sites. Sample collection and dispatch for analysis are often delayed due to sites’ remoteness and limited local laboratory facilities. The standard method of dissolved organic carbon (DOC) and total organic carbon (TOC) analysis in water samples requires storage of the samples at 4 °C after collection and analysis with a minimum of delay. However, there is no freezing storage time limit specified to avoid degradation. To overcome the limitations of this approach, we investigated the method of storage, that is refrigeration (4 °C) versus freezing (-18 °C), and the effect of storage time on DOC and TOC fractions in runoff water. Storage of samples at 4 °C for more than a week resulted in a decline of TOC and DOC concentrations in runoff water.

• Freezing unfiltered water samples immediately after collection minimized TOC losses during storage, however, it may lead to variable DOC results.
• Filtering a subsample of runoff or irrigation water immediately after collection using a 0.45 µm filter and freezing both the filtered and unfiltered samples until analysis of DOC and TOC, respectively, can minimize losses during storage.

## Graphical abstract

**Figure:**
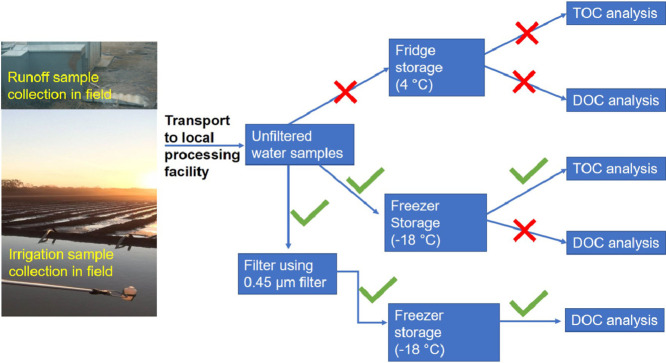

Specification table

**Table:** 

Subject Area	Agricultural and Biological Sciences
More specific subject area	*Water quality; Dissolved organic carbon*
Method name	*Storage and preservation of water samples for TOC and DOC analysis*
Name and reference of original method	APHA, 2012. Standard methods for the examination of water and wastewater, Rice et al. (Eds) 22 edition.
Resource availability	NA

## Method details

### Context

Carbon budgeting in cropping lands is gaining significance in recent years due to the introduction of the carbon pricing mechanism and carbon farming initiative where growers are provided an opportunity to trade carbon credits to offset the greenhouse gas emission from fossil fuels [1]. This warrants accurate measurements of carbon losses in various pathways. Previous studies have assumed that the major pathway of soil carbon loss is microbial respiration [2, 3]. Carbon losses through runoff and erosion at the farm-scale level were not previously focused on, but recent studies have highlighted their significance in carbon cycling [4]. Carbon losses associated with runoff and erosion in sugarcane farming systems in Australia were reported to be 12 to 44% of a sequestration rate of 0.1 t ha^−1^ yr^−1^ in the surface 10 cm of the soil [5]. In contrast, the carbon gains through irrigation water were reported to be 5 to 24% of long term carbon sequestration rates in cotton cropping systems [6]. Accurately quantifying carbon losses in runoff and erosion is, thus, of critical importance in understanding the carbon cycle in various farming systems around the world.

Runoff monitoring experiments are often conducted in remote sites where access is limited during extreme weather events, therefore dispatch to the analytical laboratory is often delayed. To accurately quantify carbon losses in runoff water, selecting and adopting appropriate methodology is essential. Sample collection, transport, storage method, storage duration, analytical method, and quality control are all vital components to ensure accurate determination of organic carbon in water samples.

The standard method widely used for dissolved organic carbon (DOC) and total organic carbon (TOC) measurements in water samples is the heated (116–130 °C) persulfate oxidation procedure following American Public Health Association Method [7]. The sample collection and preservation protocol for this analysis recommend, if possible, immediate analysis (to prevent the interference of preservatives) or storage at 4 °C immediately after collection until analysis with a maximum storage time of 7 days. However, there is no freezing storage time limit specified to avoid any changes or losses during storage. Previous studies that investigated the effect of storage method and time focused only on long term storage (138–1082 days) [8], Whereas short term (30 to 45 days) storage and preservation has not been previously assessed. To overcome limitations of this approach, we investigated the effect of storage before processing (cool room storage at 4 °C versus freezing) and storage time (cool room storage) on DOC and TOC fractions in runoff water.

### Water samples storage method comparison

The water sample collection and equipment used were described by Nachimuthu et al. [6]. This investigation was carried out using a subset of water samples. The irrigation and runoff samples were collected from two irrigation events within a cotton field. Fourteen water samples (71% runoff samples and 29% irrigation samples) (7 samples taken on 8^th^ Dec 2015 and 7 samples taken 31^st^ Dec 2015) were assessed to see if freezing would affect the ratio of TOC: DOC. Two sets of subsamples were taken with one set of samples stored in the refrigerator (4 °C) and another set of samples stored in the freezer (−18 °C) immediately after collection. The samples were transported to the laboratory and analyses conducted for both TOC and DOC on 21^st^ January 2016. The water samples were filtered using 0.45 µm filters before analysing for DOC (potential contamination of samples were avoided by using filters that did not contain any dissolved carbon fractions). Prior to analysis, inorganic carbon was purged by acidifying the sample to a pH < 2, causing the carbonates and bicarbonates present to dissociate to carbon dioxide. Both total and dissolved organic carbon were analysed using heated (116–130 °C) persulfate oxidation following the American Public Health Association Method [7]. The TOC was analysed using unfiltered samples. This paper focuses on the storage method and timing. The detailed laboratory analysis is described by Nachimuthu et al. [6]. Total Suspended Solids (TSS) were measured by filtering up to 100 mL of a well-mixed sample through a pre-weighed standard 47 mm diameter glass fiber filter (Macherey-Nagel MN GF-3) and calculating the increase in weight of the filter after drying at 105 °C for a minimum of 24 h.

### Storage method validation

**Fig. 1:**
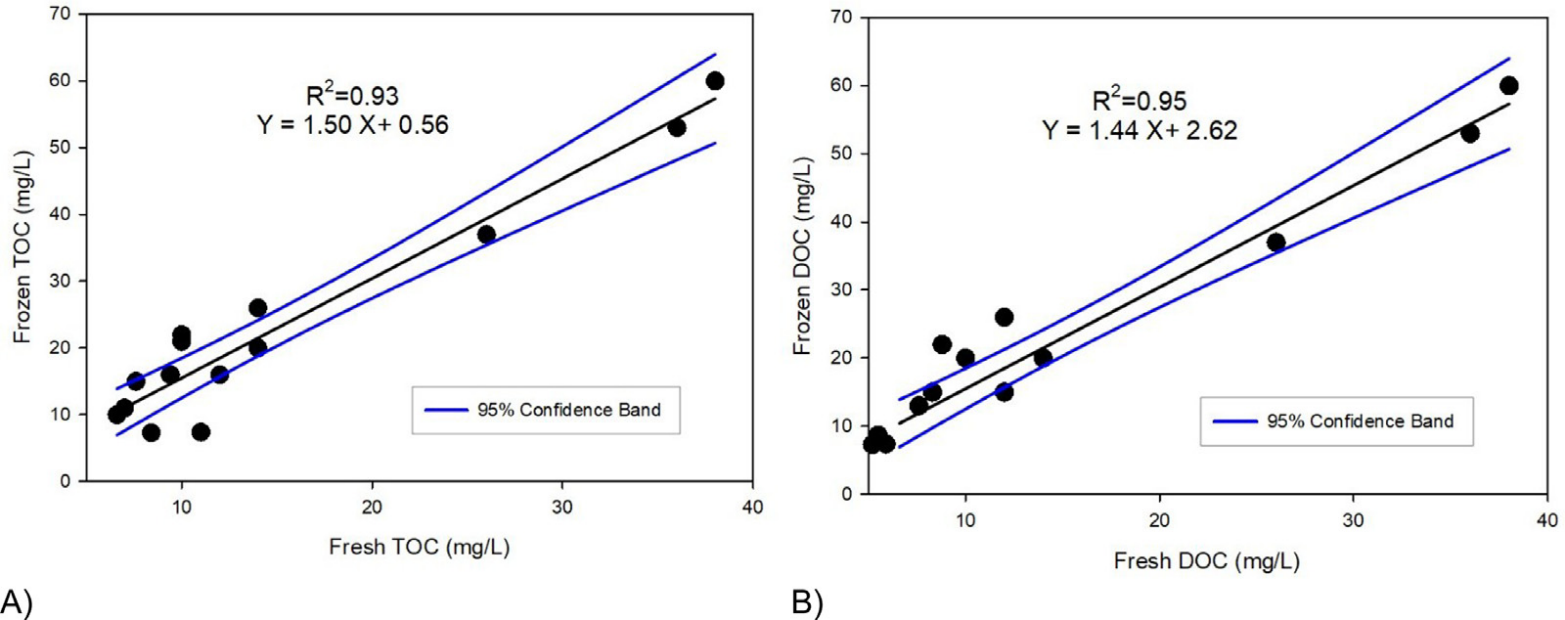
Relationship between (A) TOC (mg/L) and (B) DOC (mg/L) of frozen and unfrozen (fresh) samples. Fresh samples were stored at 4 °C and frozen samples at −18 °C.

The relationship between frozen and cold storage samples were tested using linear regression. The storage method validation results indicate that frozen TOC and DOC values were generally higher than unfrozen sample TOC concentrations (Fig. 1) suggesting a degradation or loss mechanisms occurring during 4 °C storage. The DOC as a fraction of TOC generally differed when frozen, but the effect was inconsistent. The DOC varied from 86 to 100% of TOC within frozen samples and 53 to 100% of TOC within fresh samples. TSS concentration doesn't explain this inconsistent effect (supplementary figure 1 and 2). However, there was clear evidence that freezing and thawing (frozen samples were left overnight at room temperature (~20 °C before analysis or filtering) affected DOC concentrations. We suggest, therefore, that water samples should be filtered for DOC analysis before storage in the freezer and unfiltered samples used for TOC analysis should be frozen immediately after collection. Storing the water samples in the refrigerator (4 °C) lead to a decline in both TOC and DOC concentrations. A confirmatory study was also conducted on the effect on storage time on the decline in TOC and DOC concentrations as described below.

### Water samples storage time comparison

In this study, seven samples (two irrigation and five runoff water samples) were collected during March 2016 and transported to the laboratory for analysis. The seven samples were analysed for TOC and DOC on days 1, 12, 20 and 27 after the arrival at the laboratory. Storage temperature was maintained at 4 °C. The percentage decline in concentrations were calculated and presented in Table 1 and 2.

**Table 1:** Total Organic Carbon (TOC) concentrations (mg/L) in runoff water samples over time.

Sample	1	2	3	4	5	6	7
Day 1	18	16	45	19	76	74	24
Day 12	17	15	40	18	46	71	23
Day 20	15	13	36	16	41	68	21
Day 27	17	14	31	18	34	67	20
% decline	6	13	31	5	55	9	17

**Table 2:** Dissolved Organic Carbon (DOC) concentrations (mg/L) in runoff water samples over time.

Sample	1	2	3	4	5	6	7
Day 1	15	12	40	17	48	69	19
Day 12	13	11	34	15	40	68	17
Day 20	11	9.4	30	13	37	64	16
Day 27	14	11	31	15	31	63	18
% decline	7	8	23	12	35	9	5

### Storage time validation

**Fig. 2:**
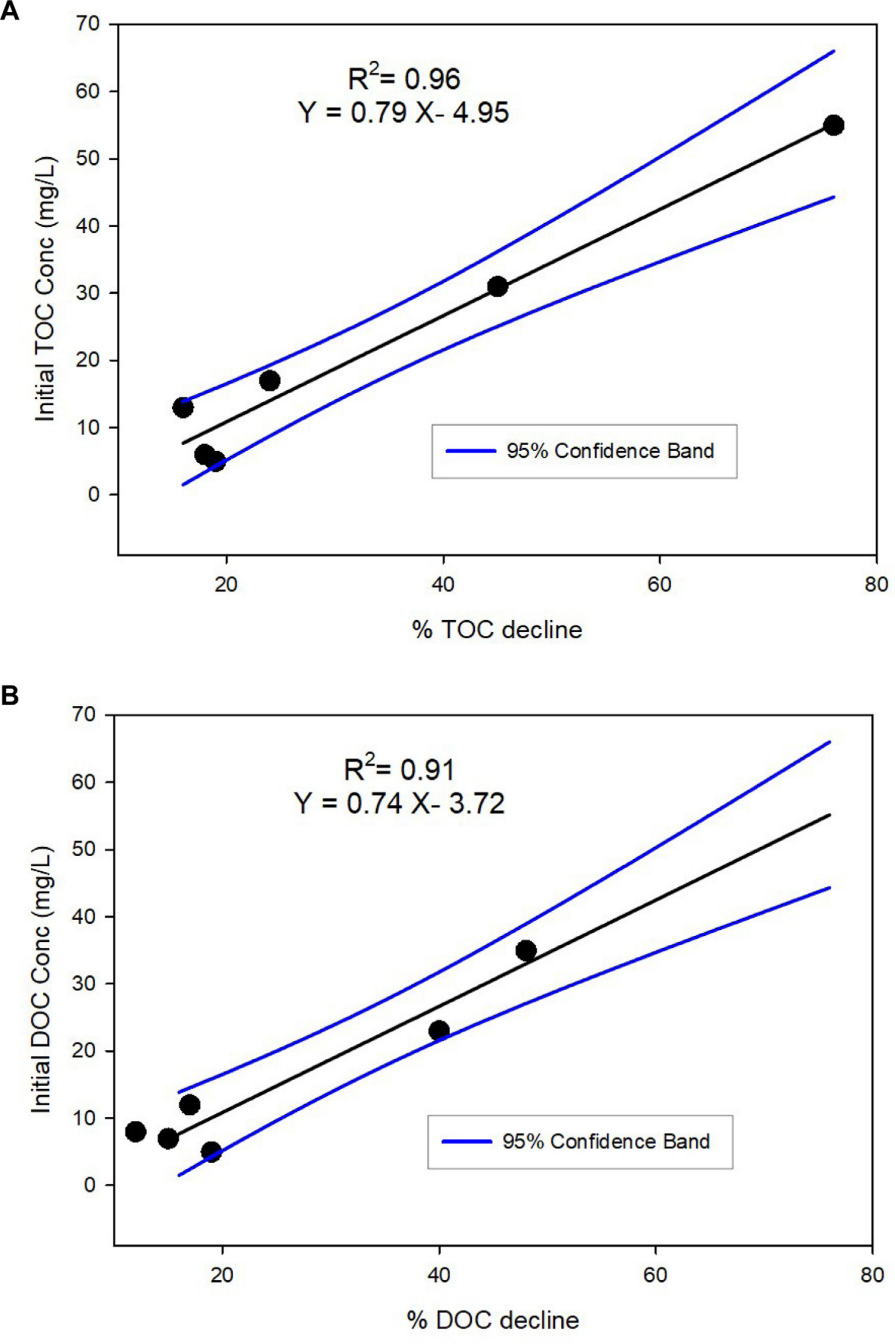
Relationship between (A) TOC (mg/L) and (B) DOC (mg/L) and % decline over time.

The storage time study suggested that there was a decline in both TOC and DOC concentrations (Table 1). However, there were variations in the magnitude of decline over time among the samples tested. Sample 6 appeared to be an outlier, and was, therefore, excluded to derive a better relationship between initial concentrations of TOC and DOC, and % decline in TOC and DOC, respectively (Fig. 2A and 2B). There was a linear relationship between initial TOC concentration and % TOC decline (R^2^ = 0.96), and initial DOC concentration and % DOC decline (R^2^ = 0.91). TSS concentrations of water samples had no influence on the level of TOC or DOC decline. The results indicated that up to 55% of TOC (Table 1) and 35% of DOC (Table 2) losses occurred during storage at 4 °C for some samples. These results suggest that when water sample collection occurs in remote areas and transport to analytical facilities is likely to be delayed, TOC and DOC losses can occur. Sample processing and storage of high quality are, thus, essential for accurate determination of TOC and DOC.

## Conclusion

The method discussed provides for better accuracy than simple refrigeration when determining TOC and DOC analysis in runoff water samples where there are delays in the order of days or weeks between collection and analytical determination. Based on the results of this study, we recommend that runoff water samples should be frozen immediately after collection for TOC measurements. For DOC analysis a subsample should be filtered as soon as possible after collection with filters that are free of any dissolved carbon components to avoid contamination followed by freezing. Storing samples in the refrigerator (4 °C), lead to a decline in both DOC and TOC concentrations, and thus, underestimated carbon losses in runoff water samples. Our findings provide guidance on storage and initial processing of runoff water samples that were not explicitly defined in previously published methods. We believe that the methodology reported in our study will improve the reliability and consistency of TOC and DOC values reported in the literature and assist editors and reviewers to identify data quality issues when samples have been stored over long periods prior to analyses.

## Declaration of Competing Interest

None

## Appendix Supplementary materials

Image, application 1
