# Supplementary material for: Storage and initial processing of water samples for organic carbon analysis in runoff
Source: MethodsX. 2020 Jul 26;7:101012. doi: 10.1016/j.mex.2020.101012 (PMC7414007; doi:10.1016/j.mex.2020.101012)
Supplement: Supplementary file 1 [file mmc1.docx]

**Supplementary material *and/or* Additional information:**

| 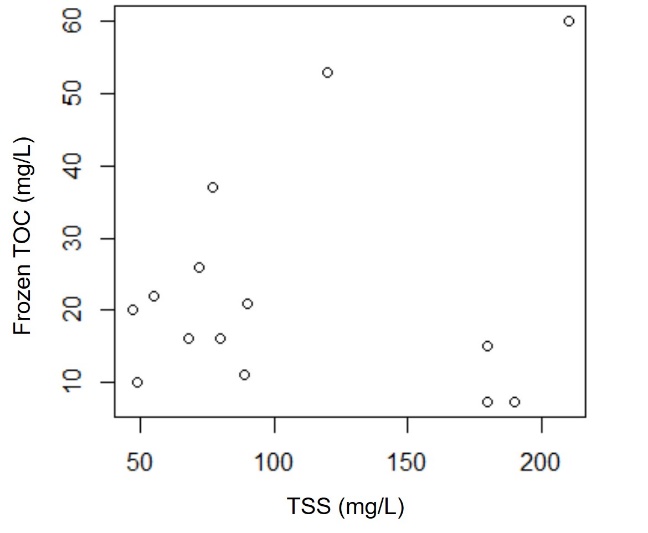 | 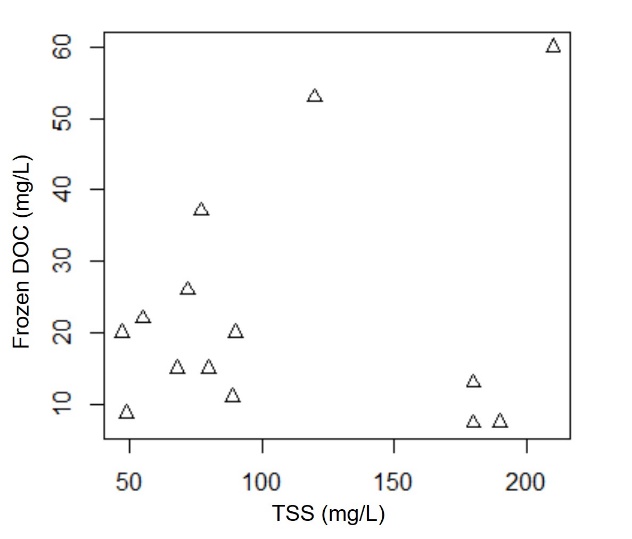 |
| --- | --- |

**Supplementary figure 1**. Effect of total suspended solids (TSS (mg/L) concentrations on TOC (mg/L) and DOC. There is no relationship between TSS and TOC or DOC.
